# Assessment of Myocardial Perfusion at Rest and During Stress Using Dynamic First-Pass Contrast-Enhanced Magnetic Resonance Imaging in Healthy Dogs

**DOI:** 10.3389/fvets.2018.00211

**Published:** 2018-09-04

**Authors:** Henning Richter, Patrick R. Kircher, Fabiola B. Joerger, Erika Bruellmann, Matthias Dennler

**Affiliations:** ^1^Clinic for Diagnostic Imaging, Vetsuisse Faculty, University of Zurich, Zurich, Switzerland; ^2^Division of Anesthesiology, Vetsuisse Faculty, University of Zurich, Zurich, Switzerland; ^3^Philips AG, Zurich, Switzerland

**Keywords:** myocardial perfusion, adenosine, dog, heart, dynamic contrast-enhanced magnetic resonance imaging

## Abstract

**Objective:** To assess the feasibility of myocardial perfusion analysis in healthy dogs using dynamic contrast-enhanced cardiac magnetic resonance (DCE-MR) imaging at rest and during simulated stress with two doses of adenosine.

**Animals:** Ten healthy beagle dogs.

**Procedures:** Dogs were anesthetized and positioned in dorsal recumbency in a 3.0 Tesla MR scanner. Electrocardiogram-triggered dynamic T1-weighted ultrafast gradient echo images of three slices in short-axis orientation of the heart were acquired during breath holds and the first pass of gadolinium contrast. Image acquisition was performed after 4 min infusion of 140 μg/kg/min and 280 μg/kg/min adenosine and, after a washout period, without adenosine, respectively. Images were processed by dividing each slice into 6 radial segments and perfusion analysis was performed from signal intensity-time data.

**Results:** No differences in perfusion parameters were found between segments within any of the slices, but significant differences were found between slices for peak enhancement, accumulated enhancement, and the maximum upslope. In addition, significant differences were found within each slice between data at rest and during adenosine-induced stress for the relative and absolute maximum upslope, relative peak enhancement, time to peak, and accumulated enhancement although inter-individual variation was large and no difference was found between the two stress tests for some parameters.

**Conclusion and Clinical Relevance:** Results of this study showed that rest and stress myocardial perfusion can be assessed using DCE-CMR in dogs using the methods described. Both, adenosine dose and slice appear to affect perfusion parameters in healthy dogs and individual response to adenosine was variable.

## Introduction

Cardiac magnetic resonance (CMR) imaging is a widely used modality to assess myocardial tissue and perfusion in people and has been used in a limited number of studies in dogs and cats (1–5). The most common contrast-enhanced techniques used are DGE-CMR(3, 6–11) for detecting myocardial fibrosis and stress/rest DCE-CMR (12–16) for detecting myocardial ischemia. Recent studies show that DCE-CMR compares favorably or superiorly with single photon emission computed tomography (SPECT) and positron emission tomography (PET) imaging, has fewer artifacts, superior spatial resolution and is free from ionizing radiation compared to SPECT and is not limited to sites with a cyclotron (12, 14, 17, 18).

First-pass DCE-CMR measures contrast enhancement during the first pass of a gadolinium bolus through the cardiac chambers and myocardium using fast imaging techniques. To track the first pass, dynamic image acquisition is performed at the same point in the cardiac cycle during successive heart beats, such that cardiac motion is essentially frozen. The acquisition is thus a motion-free dynamic series of cross-sectional images in which signal intensity increases and falls over time as the bolus of contrast passes through myocardial tissue(19, 20). Resting images are used to visualize regions of myocardial hypoperfusion, such as a corresponding coronary artery infarction. Ischemia associated with arterial stenosis is identified by the presence of myocardial hypoperfusion under vasodilator stress (21–24). This is most commonly achieved using adenosine, which induces vasodilation by activating Adenosine A2 receptors, leading to smooth muscle relaxation (25). As adenosine induces vasodilation in healthy but not in stenotic arteries, redistribution of blood toward non-stenotic regions from areas supplied by a stenotic vessel (“vascular steal”) allows detection of low signal areas in hypoperfused myocardium during the first passage of the contrast agent (26). Previous canine studies have used invasive intracoronary adenosine (10, 15), but non-invasive studies have not been performed in dogs and semi-quantitative or quantitative analyses are lacking. Although coronary artery disease is of minor importance in dogs, DCE-CMR may be of value in the diagnosis and characterization of other disorders that may affect myocardial perfusion or result in secondary infarction, such as cardiomyopathies, myocarditis, and cardiac and para-cardiac neoplasia (26–29). Limitations of DCE-CMR for the use in clinical practice are the need of breath holds during examination, the relative complex and time consuming examination, possible adverse effects of pharmacologic stress agents as well as relative high costs (19).

The aims of the present study were to assess stress and resting myocardial perfusion using two rates of intravenous infusion of adenosine (140 and 280 μg/kg/min) and attain semi-quantitative DCE-CMR myocardial perfusion data in the short-axis orientation (basal, mid-ventricle, and apical slices) in healthy beagle dogs. The hypotheses were that myocardial perfusion is increased by adenosine infusion using this method and is not significantly different between the slices or between adenosine infusion rates.

## Materials and methods

### Animals

Ten adult research beagle dogs (5 males and 5 females) were used in the study. Mean ±SD age was 4.6 ± 0.2 years (range, 4.4 to 4.8 years) and mean body weight was 14.1 ± 2.5 kg (range, 10.0 to 17.3 kg). All dogs were considered healthy based on physical examination and unremarkable results of a complete blood count and serum biochemistry panel. The study was approved by the Cantonal Veterinary Office (License number ZH001/15) in compliance with the Animal Welfare Act of Switzerland.

### Anesthesia

Dogs were sedated with butorphanol^1^ (0.2 mg/kg, IM) following a 12-h fast. Anesthesia was induced with propofol^2^ (4–6 mg/kg, IV) and maintained with sevoflurane^3^ (oxygen/air: 1/1) following endotracheal intubation. Dogs were mechanically ventilated in volume-controlled mode (10–15 ml/kg) with respiratory rate adjustment to achieve an end-tidal CO_2_ of 35–42 mmHg (4.66–5.59 kPa). A 22-gauge cannula was inserted in the left and right cephalic vein for administration of gadolinium and intravenous crystalloids, and adenosine, respectively. Lactated Ringer's solution (5 ml/kg/h) was administered throughout anesthesia. Anesthesia monitoring included heart rate, pulse rate, respiratory rate, invasive arterial blood pressure, oxygen saturation, inspired- and expired gases and end-tidal CO_2_. Monitored anesthesia data is part of a collateral study and is only partially included in this manuscript.

### Imaging protocol

The DCE-CMR imaging was performed with a 3.0 Tesla MRI scanner with the integrated digital whole-body coil^4^. The dogs were positioned in dorsal recumbency with the head toward the gantry. Four MRI-safe electrodes^5^ were attached on both sides of the chest wall to trigger the MR acquisition using a wireless vector cardiography unit^6^. All images were acquired during an end-expiratory breath hold to minimize respiration-induced movement of the heart. This was achieved by turning off the ventilator prior to contrast administration and following hyperventilation to reach an end-tidal CO_2_ of 35 mmHg. Breath hold controlled respiration until signal intensity of the myocardium, monitored on real time view, decreased. Mechanical ventilation was resumed following the second pass of contrast agent in-between each set of imaging studies.

**Figure 1:**
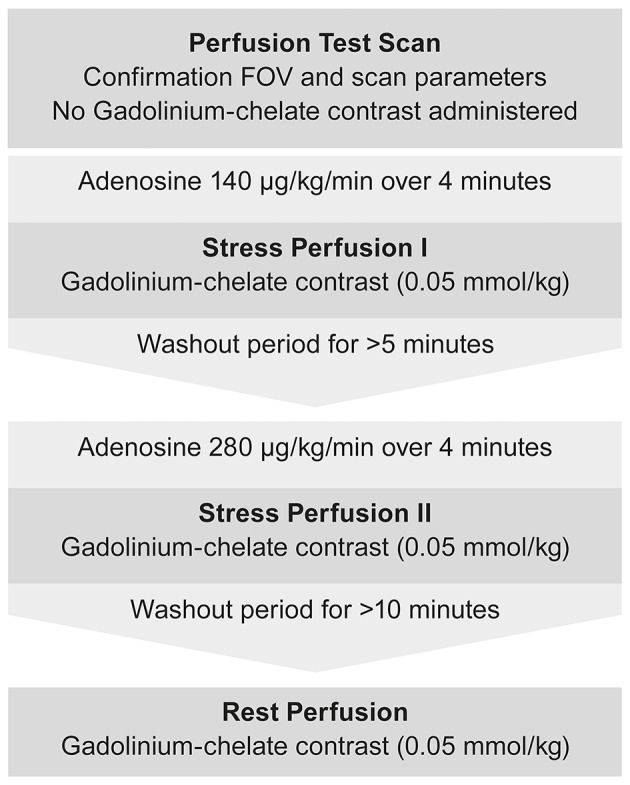
Flow chart depicting the imaging protocol used for DCE-CMR during stress and rest examination.

Initial scout images were performed to determine cardiac geometry. An initial perfusion test scan (without contrast administration) was performed to assess the FOV and confirm that sequence parameters yielded an artifact-free representation of perfusion. Subsequently, intravenous adenosine was administered at a rate of 140 μg/kg/min for the first stress perfusion measurements (S1). Image acquisition and contrast injection began after 4 min of adenosine infusion using 0.05 mmol/kg gadolinium contrast agent^7^ administered at a rate of 5.0 ml/s, followed by a 12 ml saline flush at the same rate, with a programmable syringe pump^8^. The second stress perfusion measurements (S2) were performed in the same way using intravenous adenosine at a rate of 280 μg/kg/min. A break of at least 5 min between S1 and S2 ensured independent measurements and limited the risk of carried over effects caused by late adenosine effects. The protocol prevented pharmacokinetic interactions between these examinations because adenosine has a very short biological half-life time (<10 s in human plasma). Finally, the adenosine infusion was stopped and rest perfusion images were taken after at least 10-min washout period (Figure 1).

**Figure 2:**
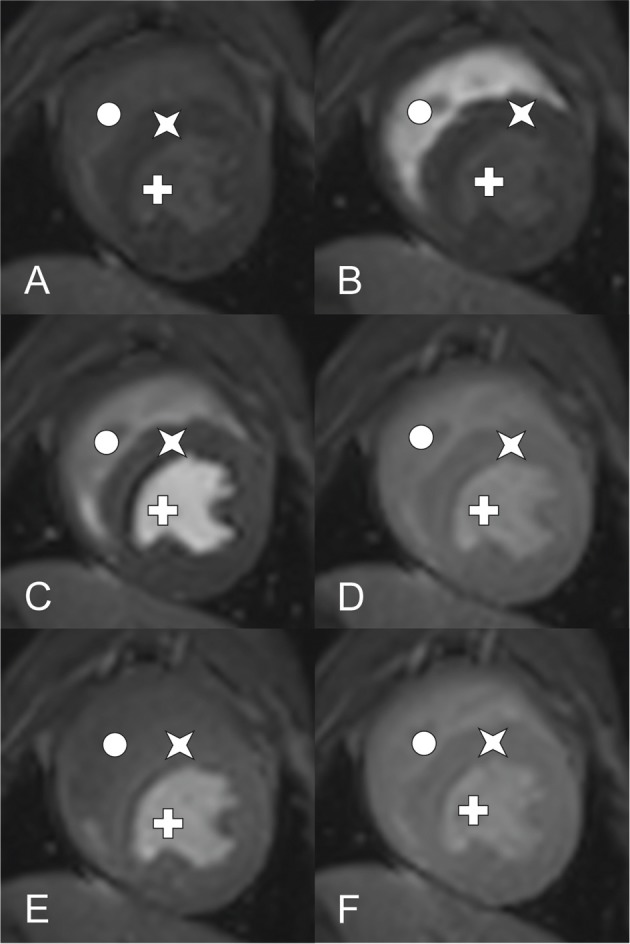
DCE-CMR of a healthy canine heart using a 3.0 Tesla MRI (Star: Myocardium, Cross: left ventricle, circle: right ventricle). Short axis view of a T1-weighted ultrafast gradient echo (Turbo field echo) images at mid ventricle level during contrast distribution. Images **(A–D)** aquired at rest perfusion, **(E)** and **(F)** at stress 1 respective stress 2 perfusion. **(A)** Early time point before contrast arrival, **(B)** contrast peak in the right ventricle, left ventricle, myocadium remain unenhanced, **(C)** contrast peak in the left ventricle, right ventricle post peak enhancement, myocadium remain unenhanced, **(D)** contrast peak in the myocardium, both ventricles post peak enhancement, **(E)** comparable time phase as **(D)** showing stress 1 perfusion, **(F)** comparable time phase as **(E)** showing stress 2 perfusion.

Dynamic T1-weighted ultrafast gradient echo (Turbo field echo) images were acquired in the short-axis orientation with one image per cardiac cycle in three left ventricular slices (base, mid-ventricle, and apical). Imaging was performed during breath hold with ECG-triggered pulse sequences using the following pulse sequence parameters: echo time, 1.7 ms; repetition time, 3.7 ms; number of signal averages, 1; flip angle, 20°; saturation prepulse delay, 100 ms; echo train length, 31 [equals phase encoding steps (SSH)]; slice thickness, 10 mm; SENSE: yes; FOV, 200 × 200 mm; matrix, 92 × 92; voxel size, 2.20 × 2.17 mm; reconstruction voxel size, 1.79 mm; reconstruction matrix, 112 (Figure 2). An example of DCE-CMR of a healthy canine heart is available as Supplementary Video.

### Image processing and analysis

**Figure 3:**
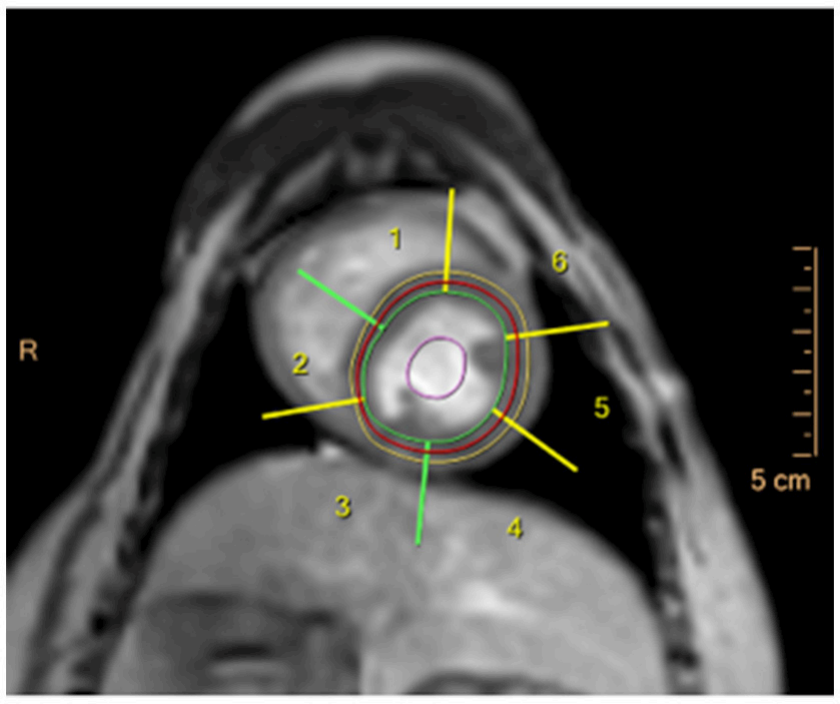
DCE-CMR image in a healthy beagle dog during adenosine infusion in the basal short-axis slice, showing the epicardial (yellow line) and endocardial (green line) contour of the left ventricle and the left ventricular myocardial segments generated by the analysis software (numbers 1–6).

**Figure 4:**
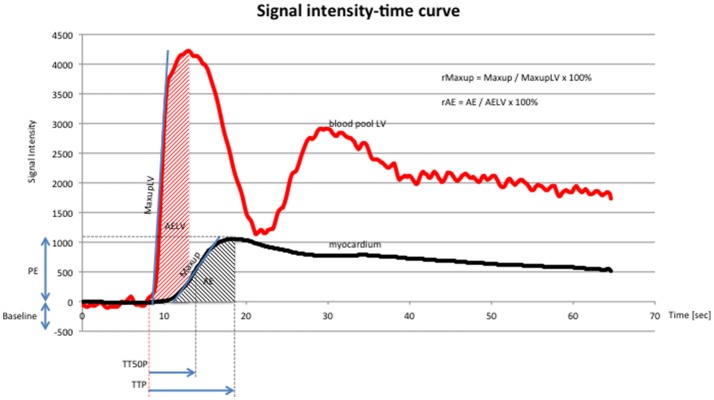
Signal intensity-time curve (x-axis: time in seconds, y-axis: signal intensity) of DCE-CMR, showing signal intensity in the blood pool (red line) of the left ventricle and the myocardium (black line). Red line: first peak reflects first pass of contrast medium, second peak reflects second pass of contrast medium. Abbreviations according to the manuscript: PE, TTP, TT50P, Maxup, r Maxup, AE, rAE. Additional abbreviations: maximum upslope of the left ventricle (MaxupLV): maximum increase in signal intensity determined by linear fit in the left ventricle; accumulated enhancement of the left ventricle (AELV): accumulated signal intensity of the left ventricle. The chosen parameters characterize the course of the curve and quantify the perfusion data.

Images were exported to a dedicated workstation^9^, which was used to assess the DICOM images using cardiac imaging analysis software^10^. Contours of the left ventricular epicardial and endocardial borders were drawn by the software and adapted manually to ensure myocardial enhancement in the stack of short-axis slices. Each slice was divided into 6 equidistant radial segments, arranged counter-clockwise starting from the anterior septal insertion of the right ventricle (Figure 3). Perfusion data analysis was performed by the software based on signal intensity-time data, whereby signal intensities from each of the six segments are plotted for each time point to generate dynamic uptake curves (Figure 4). The signal intensity-time data was analyzed with focus on parameters during the first pass of contrast medium. During distribution of contrast medium in the circulation, effects of contrast medium diffusion into myocardial interstitium influence the curve (26). The parameters are chosen to characterize the course of the intensity-time curve and to translate into numeric values. The analysis included the following parameters: peak enhancement (PE): maximum signal intensity of the myocardium normalized by the baseline signal before arrival of contrast; relative peak enhancement (rPE): maximum signal intensity relative to that measured in the left ventricle; maximum upslope (Maxup): maximum increase in signal intensity of the myocardium determined by linear fit; relative maximum upslope (rMaxup): maximum increase in signal intensity relative to that measured in the left ventricle; time to 50% peak (TT50P): time from contrast arrival in the left ventricle until 50% of the maximum signal intensity of the myocardium was reached; time to peak (TTP): time from contrast arrival in the left ventricle to maximum signal intensity; accumulated enhancement (AE): accumulated signal intensity, calculated as the area under the intensity-time curve from the start of increased signal intensity in the myocardium to the maximum signal intensity; relative accumulated enhancement (rAE): accumulated signal intensity relative to that in the left ventricle.

### Statistics

Statistical analysis was performed using a commercial software package^11^. Descriptive analyses were performed for all continuous variables. A two-way ANOVA was used to analyze the effect of segment and slice on each parameter for resting perfusion data. A Friedman test was used to analyze differences between rest, S1 and S2 pulse rates, blood pressures and perfusion data for each parameter in each slice. Where a significant difference between rest, S1 and S2 data was found, a *post-hoc* Conover analysis based on the mean rank differences of groups was performed. Significance was set at *P* < 0.05 throughout.

## Results

No complications were observed during anesthesia and all dogs recovered uneventfully. In two dogs, the R-R interval was reduced due to high heart rates and a reduced FOV was used for DCE-CMR image acquisition. No other changes were made to the sequence parameters.

The mean ±SD pulse rate was 109 ± 12, 119 ± 9, and 123 ± 14 beats/min at rest, S1 and S2, respectively. Rest pulse rates were significantly lower than S1 and S2 pulse rate but no difference was found between S1 and S2 pulse rates. The mean ±SD systolic blood pressures were 117 ± 19, 120 ± 24, and 111 ± 22 mmHg at rest, S1 and S2, respectively. The systolic pressure at S2 was significantly lower than at S1 but no difference was found between rest and either S1 or S2. The mean ±SD diastolic blood pressures were 65 ± 12, 59 ± 14, and 52 ± 12 mmHg at rest, S1 and S2, respectively. The diastolic blood pressure at S2 was significantly lower than at rest or at S1 but no difference was found between rest and S1. The mean ±SD mean arterial blood pressures were 78 ± 13, 73 ± 15, and 66 ± 13 mmHg at rest, S1 and S2, respectively. The mean arterial blood pressure at S2 was significantly lower than at rest or at S1 but no difference was found between rest and S1.

Rest perfusion data revealed no differences between segments for any of the perfusion parameters but significant differences were found between slices for Maxup, rMaxup, AE, rAE, PE, and rPE (Table 1).

**Table 1:** Perfusion dataset between slices.

**Variable**	**Slice**			***P***
	**Basal**	**Mid-ventricular**	**Apical**	
Maxup (S/s)	213 ± 57*	181 ± 50^†^	128 ± 38^‡^	< 0.001
rMaxup (%)	17.7 ± 4.9*	11.2 ± 3.3^†^	7.6 ± 2.0^‡^	< 0.001
AE (S)	9253 ± 2026*	8220 ± 1818^†^	5843 ± 1500^‡^	< 0.001
rAE (%)	69.5 ± 13.8*	48.7 ± 10.1^†^	36.7 ± 8.4^‡^	< 0.001
PE (S)	1074 ± 186*	931 ± 152^†^	675 ± 128^‡^	< 0.001
rPE (%)	36.2 ± 7.0*	23.3 ± 3.7^†^	16.0 ± 2.4^‡^	< 0.001
TT50P (s)	15.4 ± 2.3	15.6 ± 2.4	15.9 ± 2.5	0.571
TTP (s)	20.9 ± 3.6	21.2 ± 3.7	21.3 ± 3.7	0.793

*Data shown as mean ± SD*.

*,†,‡ *Values for slices with different superscripts differ significantly (P < 0.05) from each other*. *The absence of superscripts indicates that values did not differ significantly between the slices for that variable*.

Perfusion datasets at rest, S1 and S2 revealed considerable intra-individual variation and overlap of some data (Table 2). Significant differences were found between rest and stress datasets for Maxup, rMaxup, AE, and rPE, TTP and TT50P in all three slices, and for PE and rAE in at least one slice (Table 2). In addition, significant differences between S1 and S2 datasets were found for rPE, TTP and TT50P in all three slices, and for Maxup, rMaxup, PE, and rAE in at least one slice (Table 2).

**Table 2:** Perfusion dataset at rest, stress 1 and stress 2 examination.

**Variable**	**Slice**	**Sequence**			***P***
		**R**	**S1**	**S2**	
Maxup (S/s)	1	208 (193–242)*	284 (193–386)^†^	357 (192–503)^‡^	< 0.001
	2	176 (153–214)*	248 (179–336)^†^	315 (148–438)^‡^	< 0.001
	3	124 (107–159)*	174 (108–230)^†^	224 (119–339)^‡^	< 0.001
rMaxup (%)	1	17.4 (14.1–21.7)*	20.4 (11.0–24.3)^†^	31.9 (15.5–72.4)^‡^	< 0.001
	2	10.4 (8.8–12.9)*	11.7 (7.6–23.5)	15.3 (7.8–21.9)^‡^	0.007
	3	7.3 (6.1–8.5)*	10.3 (5.3–14.4)^†^	12.3 (6.9–18.7)^‡^	< 0.001
AE (S)	1	9209 (7350–10828)*	8048 (5583–10808)^†^	8440 (4385–10407)^‡^	< 0.001
	2	8042 (6652–9508)*	7269 (5109–9973)^†^	6466 (4267–9121)^‡^	< 0.001
	3	5825 (4608–6778)*	4874 (3712–6646)^†^	5437 (3773–6653)^‡^	0.003
rAE (%)	1	70.2 (63.2–78.0)	55.6 (41.6–83.7)	61.4 (51.9–121.7)	0.445
	2	46.3 (41.8–55.1)*	40.2 (36.1–48.7)^†^	38.5 (29.5–52.8)^‡^	< 0.001
	3	35.7 (31.5–41.5)	32.9 (26.9–42.6)	33.9 (26.6–44.3)	0.732
PE (S)	1	1061 (981–1203)	1184 (754–1447)	1146 (974–1383)	0.289
	2	920 (840–1034)*	978 (700–1287)^†^	1032 (862–1254)^‡^	< 0.001
	3	644 (592–786)*	669 (502–910)^†^	731 (615–1009)^‡^	< 0.001
rPE (%)	1	36.3 (30.6–41.7)*	31.5 (22.2–38.6)^†^	43.7 (35.0–83.3)^‡^	< 0.001
	2	23.1 (20.5–25.6)*	22.0 (17.5–26.3)^†^	26.4 (21.2–32.6)^‡^	< 0.001
	3	15.9 (14.3–17.8)*	17.0 (12.9–21.9)^†^	19.5 (15.1–22.9)^‡^	< 0.001
TTP (s)	1	19.7 (18.5–21.7)*	16.9 (15.7–21.0)^†^	17.3 (13.6–21.6)^‡^	< 0.001
	2	20.4 (18.5–22.5)*	18.3 (16.0–21.5)^†^	17.3 (13.8–21.3)^‡^	< 0.001
	3	20.2 (18.9–22.4)*	19.1 (16.2–21.0)^†^	18.7 (13.8–22.2)^‡^	< 0.001
TT50P (s)	1	15.0 (13.6–16.6)*	14.1 (12.5–15.3)^†^	13.1 (10.9–15.6)^‡^	< 0.001
	2	15.0 (13.9–16.8)*	14.1 (12.5–15.6)^†^	13.5 (10.9–16.0)^‡^	< 0.001
	3	15.3 (13.9–16.8)*	14.5 (12.8–15.6)^†^	13.5 (10.9–15.6)^‡^	< 0.001

*Slice: 1, basal; 2, midventricular; 3, apical*.

*,†‡ *Values within a variable and slice with different superscripts differ significantly (P < 0.05) from each other*.

*The absence of superscripts indicates that values did not differ significantly between the sequences for that variable and slice*.

## Discussion

This study evaluated myocardial perfusion using DCE-CMR at rest and stress with two different adenosine infusion rates in healthy beagles. A variety of different methods have been used to divide the left ventricle into segments in the short-axis view for DCE-CMR in people, with numbers of segments ranging from 9 to 400, depending on the clinical or research application (30–32). The American Heart Association Writing Group recommend 6 segments in the basal and mid-ventricle slices but only 5 in the apical (33). In the present study, 6 segments were selected in all three slices such that the border between segments was continuous from the base to the apex of the heart. As no difference was found in perfusion parameters between segments within the same slice, the number and manner in which segments were chosen does not appear to affect perfusion parameters in healthy dogs. Moreover, as all other data analyses were based on the median values in all 6 segments within a slice, variations due to segment number or position were not evaluated in the present study. In DCE-CMR in people, standardization of segmentation is based on assigning individual segments to specific coronary artery territories although considerable individual variation exists (33, 34). The extent to which the DCE-CMR segments used in the present study correlate to specific arterial supply in dogs was not evaluated in the present study.

The standard three slices (basal, mid-ventricle, and apical) for the short-axis orientation used in human DCE-CMR were used in the present study (33). Likewise, a slice thickness of 10 mm used in the present study conforms to that recommended for imaging in people (33), and adequate images were obtained with this thickness.

One of the most important challenges of DCE-CMR are artifacts due to cardiac and respiratory motion (35). However, electrocardiographic gating and breath holding largely eliminate these problems. In addition, the need for general anesthesia in veterinary medicine eliminates the risk of patient motion but introduces a higher patient risk and possible alterations in cardiovascular parameters due to sedative and anesthetic agents (36, 37). This may be a serious limitation to the clinical use of DCE-CMR in dogs affected by heart disease.

The goal of simulating stress using adenosine infusion in DCE-CMR is to create maximum vasodilation of the coronary arteries. Potential adverse effects of adenosine infusions in people include bronchospasm, and transient hypotension, heart block or sinus tachycardia, and contraindications for its use include 2nd or 3rd degree atrioventricular block, hypotension, bradycardia, and active bronchoconstrictive disease (27). Nonetheless, numerous studies demonstrate that adenosine infusions can be safely administered (10, 15, 38, 39). The most frequently recommended dose in people is 140 μg/kg/min although an increase of up to 210 μg/kg/min has been recommended if heart rate does not increase and/or blood pressure does not decrease after 2–3 min of the standard rate of infusion (27). We therefore evaluated both 140 μg/kg/min and a higher dose of 280 μg/kg/min in order to ensure maximum stress simulation. As expected, both doses of adenosine lead to an increase in pulse rate compared to resting measurements. Although a decrease in blood pressure is observed in most people following adenosine infusion, this has not been consistently observed in all people and was contradictory described in previous studies in dogs (15, 21, 40, 41). The maximum activity of adenosine in dogs is described to be about 15–30 s after the injection causing a dose-dependent sinus bradycardia and a dose-dependent decreasing systemic pressure as well as an arterial and coronary vasodilation (42–44).

In the present study, only the higher dose of adenosine lead to a significant decrease in diastolic and mean arterial pressure, but no consistent decrease in systolic blood pressure was found and apparent inter-individual differences in the sensitivity to adenosine infusion were evident. Based on our results it is an unsolved question, why the effect of adenosine was not that reliable as described elsewhere. An explanation might be, that inter-individual differences of the dogs have had an influence. Beside different heart rates at rest, gender could have caused a significant interaction between gender and systolic blood pressure (45, 46). Although changes in peripheral arterial blood pressure do not correlate with coronary arterial perfusion, this finding appears to refute our hypothesis that there is no difference between adenosine doses. Moreover, rPE was found to be significantly greater using the higher dose, suggesting increased myocardial hyperemia at this dose.

The expected effects of adenosine-induced vasodilatation were a faster TTP, a steeper Maxup and a greater PE compared to resting perfusion. Although results of the present study were not statistically significant in all slices for all parameters, Maxup and PE were notably different, suggesting that adenosine-induced myocardial hyperemia can be induced in dogs using the method described in this study. Given the apparent inter-individual variation in blood pressures and perfusion data, further studies with a bigger population may be necessary to refine the method and evaluate the extent to which higher adenosine doses (280 μg/kg/min) could be used. Furthermore, clinical relevance has to be defined regarding the limitations mentioned.

The hypothesis that the choice of slice would have no influence on myocardial perfusion measurements must be rejected based on findings in this study. These suggest that signal intensity decreases from the base to the apex of the heart. This may be due to physiologic variations in coronary artery blood supply. As the arteries have their origin at the heart base, more time is necessary to overcome the distance to the apex of the heart, at which time signal intensity is lower. The difference in perfusion parameters between the slices implies that data from a region of interest must always be compared with data from the same slice.

Given different imaging techniques and methods, direct comparison of results of this study with previous studies is difficult. This is the first non-invasive study in dogs describing the feasibility of myocardial perfusion analysis in healthy dogs using DCE-MR imaging at rest and during simulated stress with two different doses of adenosine. Invasive intracoronary adenosine was already used in previous canine studies (10, 15). Studies with human patients focused more on the effect of coronary artery disease (1, 2), the assessment of diastolic function by CMR (28) or myocardial perfusion and myocardial perfusion reserve index (14, 26, 47–49).

A clear limitation of this study was the small population size, limited by ethic al considerations. As the study aimed to evaluate the feasibility of a technique, it is evident that the study population is small and can't represent the general population. Additionally, the dogs were considered to be healthy based on a physical exam and blood examination. Possible cardiac diseases, such as systolic or diastolic dysfunction secondary to intermittent arrhythmias, progressed myocarditis or primary cardiomyopathies, could not be confidently ruled out without an echocardiogram. Additionally, the DCE-CMR dataset could be influenced by lack of reproducibility (i.e., between-day variability) and repeatability (i.e., within-day variability), as this could not have taken into consideration in the study design due to ethical considerations. Although this is a limitation of the study, this variability reflects the all-day-situation of future examinations of clinical patients. A randomization of the administration of the two doses, could have been a potential improvement of the study to ensure that there was no carried over effect between the stress measurements. Based on the known short half-life time of adenosine (< 10 s in human plasma) and a break of several minutes between both stress examinations, the influence of a potential carried over effect should be negligible. As the aim of the study was to evaluate the feasibility of a new technique in dogs, an interobserver variability was not included, but could be of interest in further studies.

In conclusion, this study showed that rest and stress myocardial perfusion can be assessed using DCE-CMR in dogs using the methods described. Our results indicated that both adenosine dose and slice may affect semi-quantitative perfusion data. Further studies are necessary to evaluate the usefulness of DCE-CMR in the characterization of canine myocardial disorders.

## Author contributions

HR, PK, EB, MD: concept and study design; HR, PK, MD: animal permission; HR, EB, FJ, MD: data acquisition; HR, PK, FJ, EB, MD: data analysis; HR, FJ, EB, MD: manuscript writing; HR, PK, FJ, EB, MD: review manuscript.

### Conflict of interest statement

EB was employed by company Philips AG, Switzerland. The remaining authors declare that the research was conducted in the absence of any commercial or financial relationships that could be construed as a potential conflict of interest.

## Footnotes

^1^Morphasol, Dr. E. Graeub AG, Bern, Switzerland.

^2^Propofol, Fresenius Kabi AG, Oberdorf, Switzerland.

^3^Sevorane, AbbVie AG, Baar, Switzerland.

^4^Philips Ingenia 3.0T with a dStream anterior/posterior body coil, Philips AG, Zurich, Switzerland.

^5^Kendall 750 radiolucent tape electrodes, Medtronic (Schweiz) AG, Münchenbuchsee, Switzerland.

^6^Invivo Wireless ECG, Invivo Corporation, Orlando, USA.

^7^Omniscan, GE Healthcare AG, Glattbrugg, Switzerland.

^8^Accutron MR, Medtron AG, Saarbrücken, Germany.

^9^Extended MR Workspace, Philips AG, Zurich, Switzerland.

^10^Cardiac Explorer, Philips AG, Zurich, Switzerland.

^11^IBM SPSS Statistics for Windows, version 21.0, IBM Corp. Armonk, NY, USA.

## Supplementary material

Supplementary Video DCE-CMR of a healthy canine heart using a 3.0 Tesla MRI. Short axis view of a T1-weighted ultrafast gradient echo (Turbo field echo) images at mid ventricle level during contrast distribution. Contrast distribution followed blood flow. After enhancement of the right ventricle, the contrast peaked in the left ventricle and finally enhanced the myocardium. Breath-hold started with contrast arrival in the right ventricle and lasted into the wash out phase of myocardial enhancement.

## Abbreviations

AE: Accumulated signal enhancement
CMR: Cardiac magnetic resonance
DCE-CMR: Dynamic contrast-enhanced cardiac magnetic resonance
FOV: Field of view
Maxup: Maximum upslope
MRI: Magnetic resonance imaging
PE: Peak enhancement
rAE: Relative accumulated enhancement
rMaxup: Relative maximum upslope
rPE: Relative peak enhancement
S1: First stress perfusion examination
S2: Second stress perfusion examination
TT50P Time to 50% peak enhancement
TTP: Time to peak enhancement.
